# Comparison of Metabarcoding and Microscopy Methodologies to Analyze Diatom Communities in Five Estuaries Along the Southern Coast of the Korean Peninsula

**DOI:** 10.1007/s00248-024-02396-x

**Published:** 2024-07-17

**Authors:** Young-Saeng Kim, Hyun-Sik Yun, Jae-Hak Lee, Kyung-Lak Lee, Jae-Sin Choi, Doo Hee Won, Yong Jae Kim, Han-Soon Kim, Ho-Sung Yoon

**Affiliations:** 1 Department of Biology, College of Natural Sciences, Kyungpook National University, Daegu, 41566 Republic of Korea; 2 Research Institute of Ulleung-do & Dok-do, Kyungpook National University, Daegu, 41566 Republic of Korea; 3 School of Life Sciences and Biotechnology, BK21 Plus KNU Creative BioResearch Group, Kyungpook National University, Daegu, 41566 Republic of Korea; 4 Advanced Bio-Resource Research Center, Kyungpook National University, Daegu, 41566 Republic of Korea; 5 Water Environmental Engineering Research Division, National Institute of Environmental Research, Incheon, 22689 Republic of Korea; 6 Doohee Institute of Ecological Research, Korea Ecosystem Service Inc., Ansan, 15426 Republic of Korea; 7 Department of Biomedical Science, Daejin University, Pocheon, 11159 Republic of Korea

**Keywords:** Heterokontophyta, Diatom community, Microalgal, Microscopy, Illumina MiSeq, Taxonomy

## Abstract

The study of microalgal communities is critical for understanding aquatic ecosystems. These communities primarily comprise diatoms (Heterokontophyta), with two methods commonly used to study them: Microscopy and metabarcoding. However, these two methods often deliver different results; thus, their suitability for analyzing diatom communities is frequently debated and evaluated. This study used these two methods to analyze the diatom communities in identical water samples and compare the results. The taxonomy of the species constituting the diatom communities was confirmed, and both methods showed that species belonging to the orders Bacillariales and Naviculales (class Bacillariophyceae) are the most diverse. In the lower taxonomic levels (family, genus, and species), microscopy tended to show a bias toward detecting diatom species (*Nitzschia frustulum, Nitzschia inconspicua, Nitzschia intermedia, Navicula gregaria, Navicula perminuta, Navicula recens, Navicula* sp.) belonging to the Bacillariaceae and Naviculaceae families. The results of the two methods differed in identifying diatom species in the communities and analyzing their structural characteristics. These results are consistent with the fact that diatoms belonging to the genera *Nitzschia* and *Navicula* are abundant in the communities; furthermore, only the Illumina MiSeq data showed the abundance of the *Melosira* and *Entomoneis* genera. The results obtained from microscopy were superior to those of Illumina MiSeq regarding species-level identification. Based on the results obtained via microscopy and Illumina MiSeq, it was revealed that neither method is perfect and that each has clear strengths and weaknesses. Therefore, to analyze diatom communities effectively and accurately, these two methods should be combined.

**Supplementary Information:**

The online version contains supplementary material available at 10.1007/s00248-024-02396-x.

## Introduction

Heterokontophyta (Bacillariophyta), also known as diatoms, are a group of microalgae that play an important role in aquatic ecosystems [1]. Specifically, they support these ecosystems as primary producers through photosynthesis [1] and are also involved in cycling nutrients, especially carbon, nitrogen, and phosphorus [2, 3]. Consequently, diatoms can purify water contaminated with organic matter and nitrogen and phosphorus compounds [4, 5]. Furthermore, it is possible to indirectly monitor the aquatic environment by collecting information about diatom communities [6, 7]. The importance of such information has been previously recognized, and attempts have been made to monitor these microalgae [6, 8]. Microscopy is the predominant method through which analyses are performed [8]. Microscopy studies, which are based on morphology, have promoted the development of phylogenetic systematics for the taxonomic classification of diatoms [9]. However, community analyses based on morphology have many limitations [10, 11]. Although the morphological characteristics of each diatom species are relatively clear, extensive knowledge and expertise remain required for identification [10]. In addition, microscopic analysis requires considerable effort, and there is a limit to the number of samples a researcher can handle [12]. Therefore, a method that can compensate for the limitations of microscopy analysis is needed [10, 12].

Recently, molecular methods have been applied to analyze microbial communities [13], one of which is Illumina MiSeq [13], in which the 16s rRNA gene is used as a marker [14]. Sequencing environmental samples allows for a more effective analysis of the structure and characteristics of bacterial communities than microscopy [14]. Although fungi are relatively easy to classify morphologically compared with bacteria, a powerful tool is required due to the limitations of previous community analysis methods [15, 16]. Similar to bacterial communities, fungal communities can also be effectively analyzed via Illumina MiSeq [15, 16] using the 18s rRNA gene as a marker [15]. Analyzing fungal communities using Illumina MiSeq targeting the 18 S rRNA gene suggested that it could be used to explore other eukaryotic microbial organisms [15, 16]. Moreover, Illumina MiSeq has been specifically attempted in several previous studies to evaluate microalgal communities [17]. These have detected various microalgal taxa, including Heterokontophyta (Bacillariophyta) and Chlorophyta, which are part of the eukaryotic microbial community [17]. Therefore, previous studies have shown that Illumina MiSeq analysis can be used to improve our understanding of diatoms within microalgal communities [17].

An extensive knowledge of microalgae, including diatoms, is essential for a deep understanding of aquatic ecosystems [1, 6], which is useful for human society [1, 18]. For example, changes in the size and structure of microalgal communities greatly impact human life [18]. Sudden microalgal blooms caused by eutrophication represent a serious problem when using water resources [18, 19]. In particular, the emergence and dominance of toxin-producing microalgal species is a critical factor [18, 19]. In addition, issues associated with the rapid blooming of microalgae, such as red tides, disturb the aquatic ecosystem and limit the use of aquaculture and aquatic resources [20]. Therefore, accurate and rapid analyses of microalgal communities are essential to prevent and quickly respond to these challenges [20]. This study attempted to compare microscopy based on morphological classification and Illumina MiSeq analyses using the 18 S rRNA gene. Through this comparison, the strengths and weaknesses of each method were confirmed. Furthermore, strategies for conducting rapid and accurate analyses of diatom communities are discussed.

## Materials and Methods

### Sample Collection

**Fig. 1:**
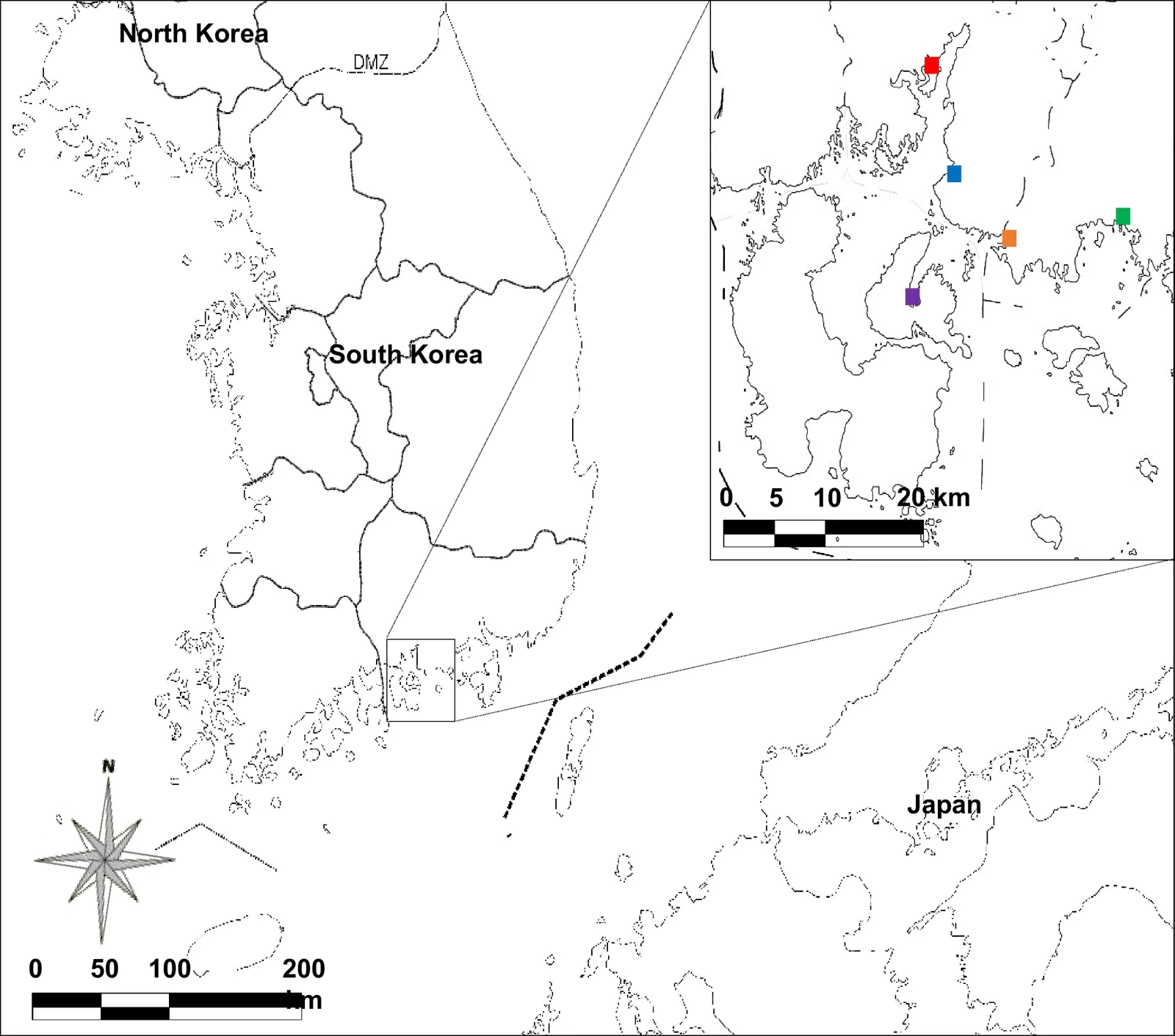
Map of the sampling sites on the southern coast of the Korean Peninsula. The locations of the sampled estuaries in Gyeongsangnam-do, South Korea, are shown in the black box. The colored square symbols indicate: Mukgok (red square, 35°03’00.8"N 128°00’44.3"E, 1172 − 340, Hwandeok-ri, Gonyang-myeon, Sacheon-si), Jangchi (green square, 34°56’36.7"N 128°14’48.1"E, 993-8, Sambong-ri, Samsan-myeon, Goseong-gun), Changseon (purple square, 34°51’56.1"N 128°00’59.4"E, 10 − 1, Sangjuk-ri, Changseon-myeon, Namhae-gun), Sagok (orange square, 34°55’17.7"N 128°07’16.3"E, 706-5, Deokho-ri, Hai-myeon, Goseong-gun), and Songpo (blue square, 34°58’14.3"N 128°03’00.4"E, 1510-14 Songpo-dong, Sacheon-si)

Water samples were collected in October 2020 from five estuaries of tributaries located in Gyeongsangnam-do, South Korea: Mukgok stream, 35°03’00.8"N 128°00’44.3"E, 1172–340, Hwandeok-ri, Gonyang-myeon, Sacheon-si; Jangchi stream, 34°56’36.7"N 128°14’48.1"E, 993–8, Sambong-ri, Samsan-myeon, Goseong-gun; Changseon stream, 34°51’56.1"N 128°00’59.4"E, 10–1, Sangjuk-ri, Changseon-myeon, Namhae-gun; Sagok stream, 34°55’17.7"N 128°07’16.3"E, 706–5, Deokho-ri, Hai-myeon, Goseong-gun; Songpo stream, 34°58’14.3"N 128°03’00.4"E, 1510–14 Songpo-dong, Sacheon-si (Fig. 1). A total of 2 L of surface water flowing toward the sea was collected from each estuary to analyze the diatom communities. The collected samples were stored in a cooler box at a low temperature and transferred to the laboratory on the day of collection.

### Microscopic Analysis

For microscopic analysis, the collected water samples were centrifuged (2,000 *g*, 2 min) to collect cells, and the cells were fixed with Lugol’s solution [21]. Then, the fixed samples were cleaned using the permanganate method and mounted using Naphrax resin (Brunel Microscopes Ltd, England) [21]. Subsequently, the pretreated samples were observed using an optical microscope (1000× magnification with oil). Based on the observations, the diatom species were identified and classified according to the identification monographs of diatoms [21, 22]. Data was collected from all valves that could be observed and identified within the samples (a minimum of 450 diatom valves).

### Illumina MiSeq Sequencing Analysis

Illumina MiSeq was performed using Macrogen (Seoul, South Korea, https://dna.macrogen.com/kor/). DNA was extracted from the water samples using the PowerSoil® DNA Isolation kit (Cat. No. 12,888, MO BIO) according to the manufacturer’s protocol [23]. The quantity and quality of the extracted DNA were assessed using PicoGreen and Nanodrop. Based on the Illumina 18 S Metagenomic Sequencing Library protocols, the obtained high-quality DNA was amplified using PCR [24]. The PCR target was the 18 S rRNA region, which was amplified using the 18 S V4 primer set: forward primer TAReuk454FWD1, 5′-CCAGCA(G⁄C)C(C⁄T)GCGGTAATTCC-3′ and reverse primer TAReukREV3, 5′-ACTTTCGTTCTTGAT(C⁄T)(A⁄G)A-3′. The target DNA fragment was approximately 420 bp long [25]. After PCR, Illumina sequencing adapters were ligated, and limited-cycle amplification was performed to add multiplexing indexes [26]. Using PicoGreen, the amplified DNA fragments were pooled and normalized. The library size was verified using the TapeStation DNA D1000 ScreenTape system (Agilent), and the sequencing data were analyzed using the MiSeq™ platform (Illumina, San Diego, USA) [27]. The raw sequencing data were demultiplexed using the index sequence, and a FASTQ file was generated for each sample. The adapter sequence was removed using SeqPurge, and sequencing errors were corrected by overlapping the areas with the correct reads [28]. The low-quality barcode sequences that did not meet the standards (i.e., read length < 400 bp or average quality value < 25) were discarded. Then, the obtained barcode sequence data were identified through a BLASTN search based on the NCBI database [29]. Based on the CD-HIT at a 97% sequence similarity level, each operational taxonomic unit (OTU) was analyzed and classified at each taxonomic level (i.e., phylum, class, order, family, genus, and species; in the case of unclassified results, the unclassified category was replaced with “-”) [30].

### Calculation and Analysis of Biological Indexes

Based on the results obtained from the microscopic and Illumina MiSeq analyses, six biological indexes, i.e., richness index (RI), diversity index (H’), dominance index (DI), evenness index (J’), trophic diatom index (TDI), and diatom assemblage index of organic water pollution (DAIpo, ) were calculated as described [31–36].

*Richness index (RI)
  $$\mathrm{RI}=\frac{\left(S-1\right)}{\mathrm{ln}\left(N\right)}$$
  *S*: total number of species
  *N*: total number of individuals

*Diversity index (H’)
  $$\mathrm{H}^\prime=-\sum\limits_{i=1}^n\left(\frac{n_i}N\right)\times\mathrm{ln}\left(\frac{n_i}N\right)$$
  *n*: number of species
  *n*_*i*_: number of individuals per species
  *N*: total number of individuals
*Dominance index (DI)
  $$\mathrm{DI}=\frac{({n}_{1}+{n}_{2})}{N}$$
  *n*_1_: number of individuals of the first dominant species
  n_2_: number of individuals of the second dominant species
  *N*: total number of individuals
*Evenness index (J’)
  $$\mathrm{J}^\prime=\frac{\mathrm{H}^\prime}{\mathrm{ln}\left(S\right)}$$
  *S*: total number of species
  H': diversity Index
*Trophic diatom index (TDI)
  $$\mathrm{TDI}=100-\left\{\left(\mathrm{WMS}\times25\right)-25\right\}$$
  WMS: weighted mean sensitivity
- $$\mathrm{WMS}=\frac{\sum\nolimits_{i=1}^{n}\left({A}_{i}{S}_{i}{V}_{i}\right)}{\sum\nolimits_{i=1}^{n}\left({A}_{i}{V}_{i}\right)}$$
-
  *A*_*i*_: abundance of species in sample (%)
  *S*_*i*_: pollution sensitivity of species (1 ≦ S ≦ 5)
  *V*_*i*_: indicator value of species (1 ≦ V ≦ 3)
*Diatom assemblage index of organic water pollution (DAIpo)
  $$\mathrm{DAIpo}=50+\frac12(\sum\limits_{i=1}^pX_i-\sum\limits_{j=1}^qS_j)$$
  $$\sum\limits_{i=1}^{p}{X}_{i}$$ 
total relative frequency of saproxenous species from 1 to p in the diatom community
  $$\sum\limits_{j=1}^{q}{S}_{j}$$ 
total relative frequency of saprophilous species from 1 to q in the diatom community

### Statistical Analysis

Individual data were compared using Student’s t-test, with P values < 0.05 considered statistically significant. All experiments were performed at least in triplicate, and all results were expressed as the mean ± standard deviation.

## Results

### Diatom Detection via Microscopy and Illumina MiSeq

**Fig. 2:**
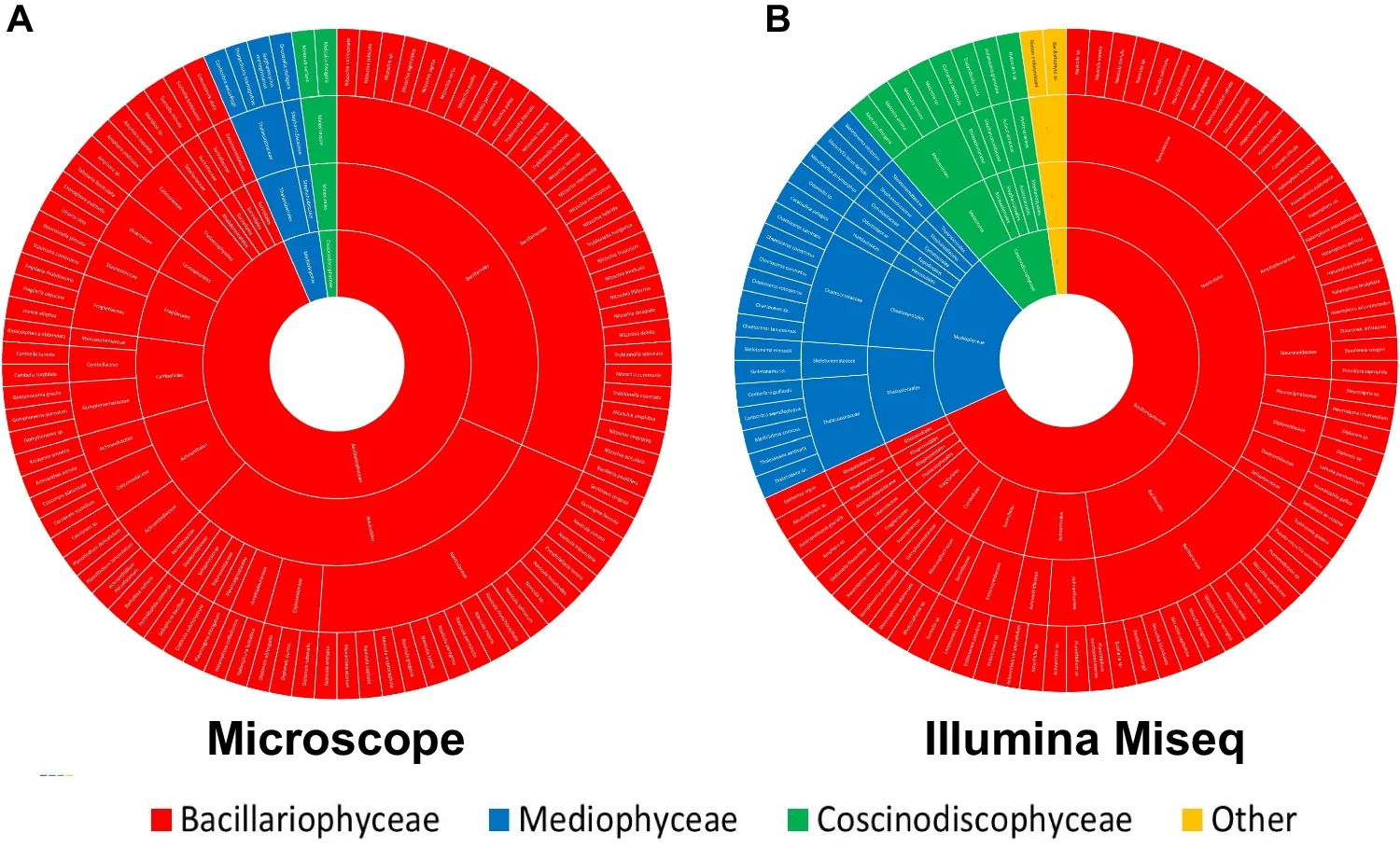
Taxonomy of the diatom species detected via microscopy and Illumina MiSeq. At the class level, the detected species belonging to Bacillariophyceae, Coscinodiscophyceae, Fragilariophyceae, and Mediophyceae are marked in red, blue, green, and purple, respectively. Species unclassified at the class level are marked in yellow. The data underlying all the diagrams indicated in this figure can be found in Table S1

**Fig. 3:**
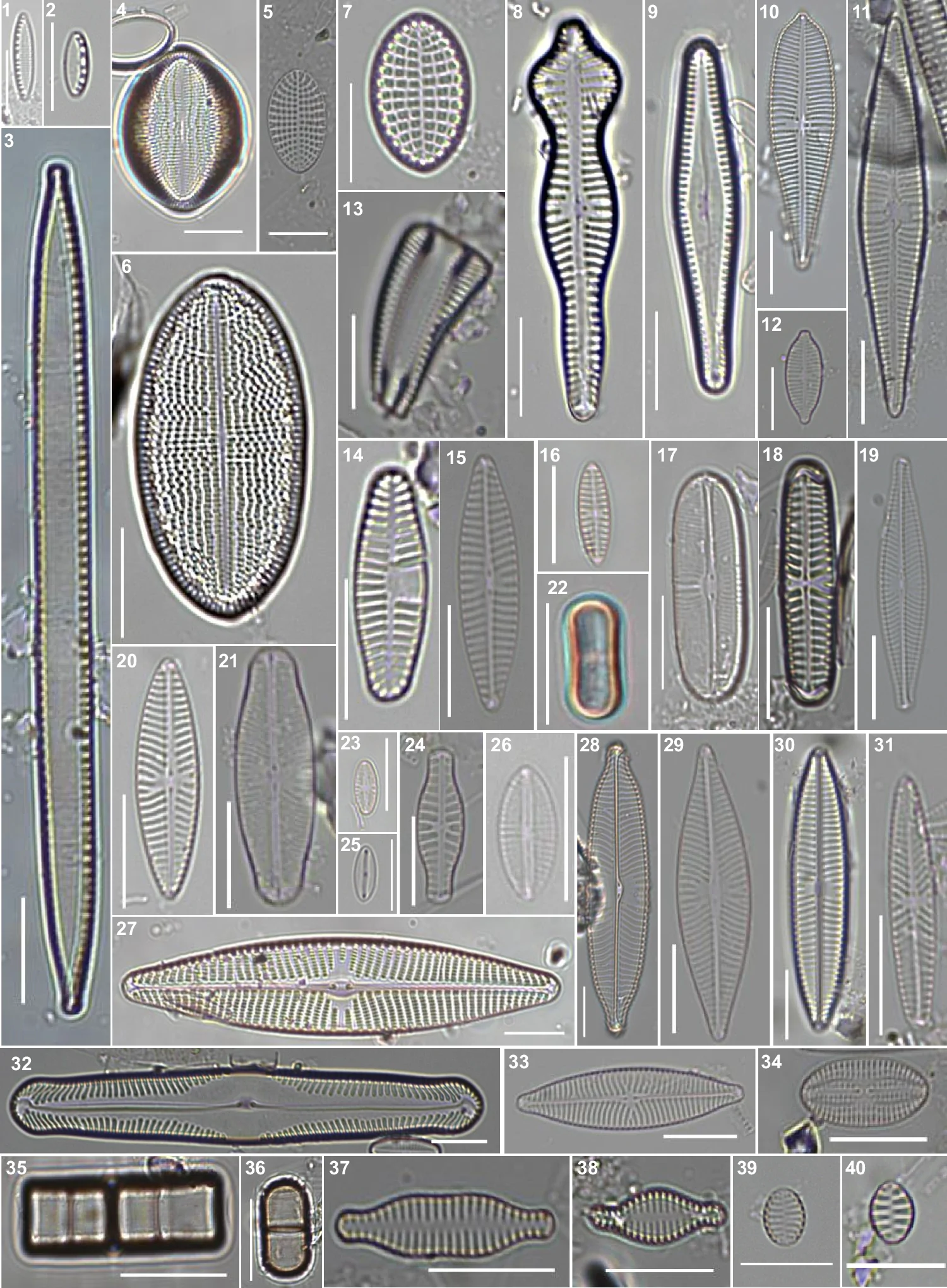
Images of diatoms obtained via light microscopy. 1–40. (1) *Nitzschia frustulum*, (2) *Nitzschia inconspicua*, (3) *Nitzschia intermedia*, (4) *Cocconeis pediculus*, (5) *Cocconeis scutellum* var. *parva*, (6) *Cocconeis lineata*, (7) *Cocconeis scutellum*, (8) *Gomphonema acuminatum* (9) *Gomphoneis clevei*, (10) *Gomphonema turris*, 11. *Gomphonema gracile*, 12. *Gomphonema parvulum*, 13. *Rhoicosphenia abbreviata*, 14. *Planothidium lanceolatum*, 15. *Navicula gregaria*, 16. *Navicula perminuta*, 17. *Sellaphora bacillum*, 18. *Hippodonta linearis*, 19. *Navicula cryptocephala*, 20. *Navicula cryptotenella*, 21. *Sellaphora pupula*, 22. *Humidophila contenta*, 23. *Navicula minima*, 24. *Navicula capitata*, 25. *Mayamaea permitis*, 26. *Craticula subminuscula*, 27. *Navicula peregrina*, 28. *Navicula viridula*, 29. *Navicula trivialis*, 30. *Navicula tripunctata*, 31. *Navicula tenelloides*, 32. *Pinnularia gibba*, 33. *Navicula zanonii*, 34. *Pseudofallacia tenera*, 35. *Melosira varians*, 36. *Melosira discigera*, 37. *Neidiomorpha binodis*, 38. *Staurosira venter*, 39. *Jousea elliptica*, 40. *Staurosirella pinnata*. Scale bars = 10 *µ*m

**Fig. 4:**
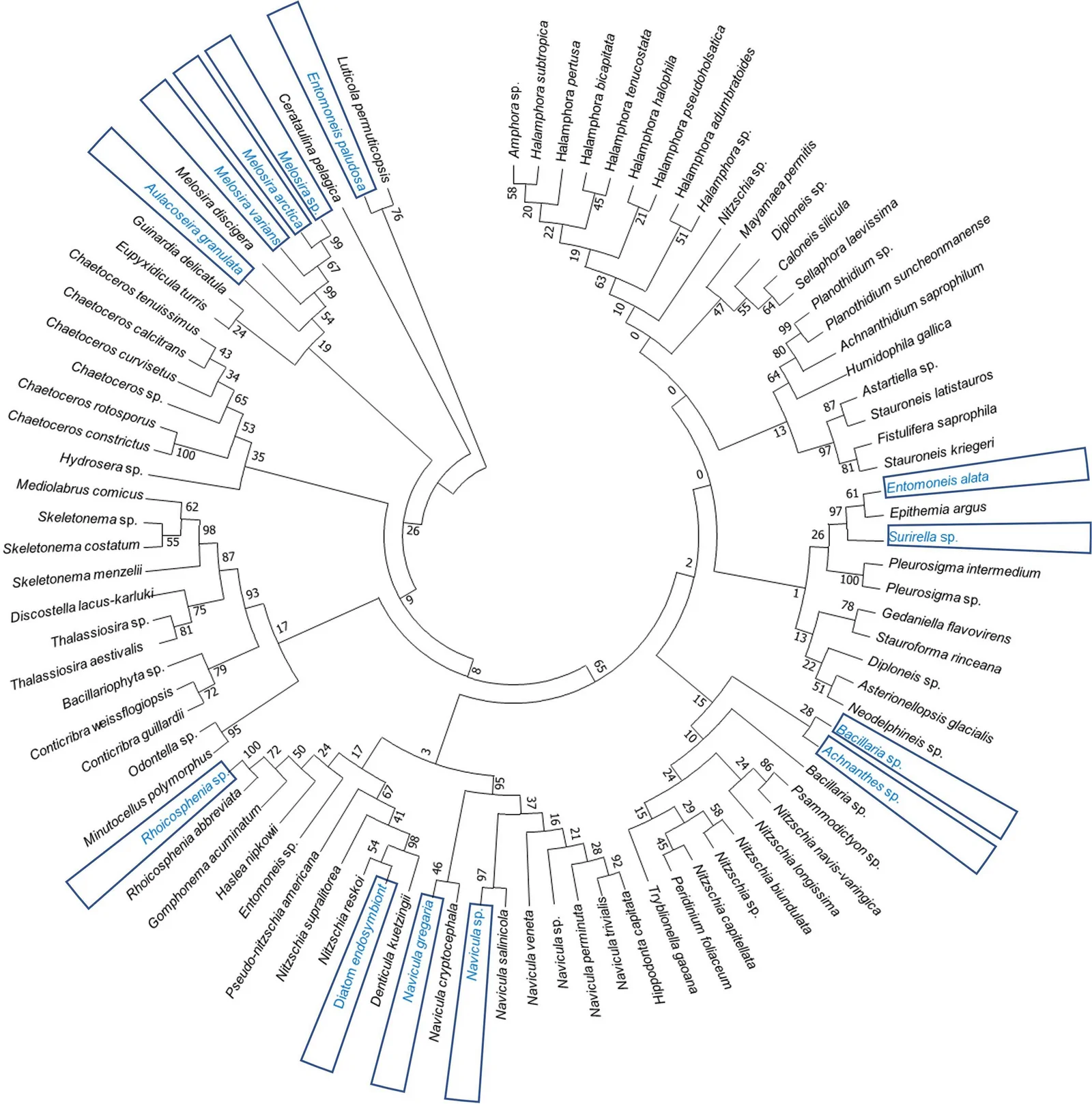
Phylogenetic tree of diatoms detected using Illumina MiSeq. Numbers at the nodes indicate bootstrap probabilities (more than 50%) for the ML analyses (1000 replicates). The dominant species found in each sample are marked with blue letters and boxes

Using microscopy and Illumina MiSeq, diatoms were identified in the water samples and classified at the phylum to species levels (Figs. 2, 3 and 4; Table S1). Microscopic analysis revealed 92 diatom species belonging to one phylum, three classes, 12 orders, and 25 families, including unclassified results. In contrast, Illumina MiSeq analysis detected 88 diatom species belonging to one phylum, five classes, 19 orders, and 32 families, including unclassified results. Among the detected species, only six (i.e., *Rhoicosphenia abbreviata*, *Navicula cryptocephala*, *Navicula gregaria*, *Navicula perminuta*, *Melosira discigera*, and *Melosira varians*) were detected in both analyses. Most of the detected diatom species at the class level belonged to Bacillariophyceae (87 and 60 species identified via microscopy and Illumina MiSeq, respectively). In addition to Bacillariophyceae, diatom species belonging to Coscinodiscophyceae (six and 22, respectively) and Mediophyceae (four and 18, respectively) were detected. The unclassified diatom species (two) at the class level were detected using only Illumina MiSeq. At the order level, ten orders belonging to Achnanthales, Bacillariales, Cymbellales, Fragilariales, Naviculales, Melosirales, Stephanodiscales, Surirellales, Thalassiophysales, and Thalassiosirales were commonly detected in both analyses. Diatom species belonging to Licmophorales and Rhabdonematales were detected only by microscopic analysis, and diatom species belonging to Achnanthales, Bacillariales, Cymbellales, Fragilariales, Naviculales, Melosirales, Stephanodiscales, Surirellales, and Thalassiosirales were detected only by Illumina MiSeq analysis. In addition, each result included unclassified species that were present at the order level in both analyses (i.e., one in microscopy and six in Illumina MiSeq). Notably, the number of diatom species belonging to Bacillariales and Naviculales was the highest in both analyses (microscopy and Illumina MiSeq). Moreover, 10 families were detected by both methods. Only microscopic analysis could detect the diatoms of 11 species. Only Illumina MiSeq analysis could detect the diatoms of 32 species, including 3 unclassified species at the family level. Both methods revealed that the numbers of diatom species belonging to Bacillariaceae (microscopic: 29 species; Illumina MiSeq: 12 species) and Naviculaceae (microscopic: 20 species; Illumina MiSeq: 12 species) were the most abundant.

### Analysis of Diatom Communities via Microscopy and Illumina MiSeq

**Fig. 5:**
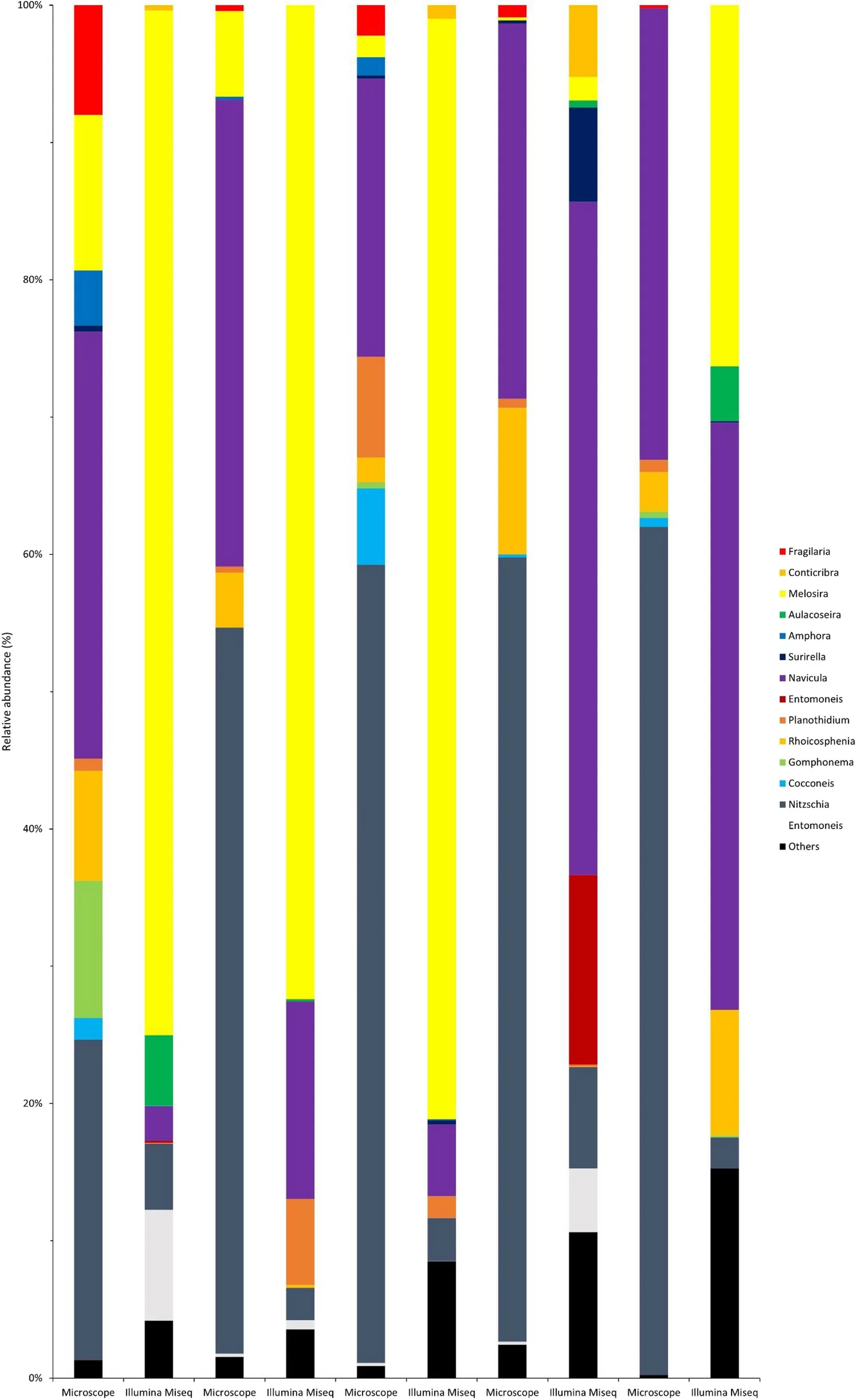
Composition of the diatom communities at the genus level for each sample analyzed via microscopy and Illumina MiSeq, showing the relative abundance of the detected diatom genera. Genra (*Bacillaria, Denticula, Psammodictyon, Pseudo-nitzschia, Tryblionella, Achnanthidium, Astartiella, Cymbella, Planothidium, Naviculales, Amphipleura, Halamphora, Diadesmis, Luticola, Diploneidaceae, Diploneis, Caloneis, Fistulifera, Haslea, Hippodonta, Mayamaea, Gyrosigma, Pleurosigma, Sellaphora, Stauroneis, Epithemia, Chaetoceros, Stephanopyxis, Guinardia, Skeletonema, Discostella, Cyclotella, Minidiscus, Thalassiosira, Asterionellopsis, Gedaniella, Meridion, Stauroforma, Synedra, Neodelphineis, Hydrosera, Minutocellus, Odontella, Cerataulina*) with relative abundance less than 5% were categorized in the Others group. The data underlying all the diagrams shown in this figure can be found in Table S1

The structural characteristics of the diatom communities at the genus level based on the results of microscopic and Illumina MiSeq analyses are presented in Fig. 5. In the five estuaries examined, the genera with a relative abundance of more than 5% detected via microscopy and Illumina MiSeq, respectively, were as follows: Mukgok (microscopic analysis: six genera, 91.78%; Illumina MiSeq analysis: three genera, 87.84%), Jangchi (microscopic analysis: three genera,93.11%; Illumina MiSeq analysis: three genera, 93.06%), Changseon (microscopic analysis: four genera, 91.11%; Illumina MiSeq analysis: two genera, 84.78%), Sagok (microscopic analysis: three genera, 95.11%; Illumina MiSeq analysis: five genera, 79.20%), Songpo (microscopic analysis: two genera, 94.67%; Illumina MiSeq analysis: four genera, 81.08%). According to the microscopy analysis results, diatoms belonging to nine genera (i.e., *Nitzschia, Gomphonema, Rhoicosphenia, Planothidium, Navicula, Melosira*, and *Staurosirella*) were dominant in the sampled communities. Moreover, in all samples, diatoms belonging to the genera *Nitzschia* and *Navicula* were dominant, and the sum of their relative abundances exceeded 50%. According to the Illumina MiSeq analysis results, 11 genera were dominant in the sampled communities. In Jangchi, Changseon, and Songpo, diatoms belonging to the genera *Navicula* and *Melosira* were dominant, whereas *Entomoneis*, *Melosira*, and *Navicula* were dominant in Mukgok and Sagok. The sum of the relative abundances of the dominant genera exceeded 60%. Differences were detected between the results obtained using the two methods. Among the major genera detected (nine and 11 were detected via microscopy and Illumina MiSeq, respectively), *Nitzschia*, *Rhoicosphenia*, *Achnanthes*, *Navicula*, and *Melosira* showed a relative abundance of more than 5%. However, according to microscopic analysis, the predominant genera with the highest relative abundance in all samples were *Nitzschia* and *Navicula*, whereas according to the Illumina MiSeq analysis, they were *Navicula* and *Melosira*. Simultaneously, some Illumina MiSeq analysis results showed that *Navicula*, *Melosira*, and *Entomoneis* were highly abundant.

Table 1 summarizes the results obtained at the species level. Both methods could detect 13 diatom species with more than 5% relative abundance. Specifically, the following abundances > 5% were observed via microscopy: *Nitzschia frustulum*, *Nitzschia inconspicua*, *Nitzschia intermedia*, *Gomphonema* sp., *Rhoicosphenia abbreviata*, *Planothidium lanceolatum*, *Navicula gregaria*, *Navicula perminuta*, *Navicula recens*, *Navicula* sp., *Melosira discigera*, and *Staurosirella pinnata*. The following abundances > 5% were observed via Illumina MiSeq in one or more samples: Diatom endosymbionts, *Entomoneis paludosa*, *Bacillaria* sp., *Rhoicosphenia* sp., *Achnanthes* sp., *Entomoneis alata*, *Navicula gregaria*, *Navicula* sp., *Surirella* sp., *Aulacoseira granulata*, *Melosira arctica*, *Melosira* sp., and *Melosira varians*. Clear differences were detected between the results obtained from the two methods regarding the composition of dominant species, and there was no significant correlation.

**Table 1:** Taxonomy and relative abundance of dominant species in the Heterokontophyta community from the five estuaries

Method	Taxonomy					Relative abundance (%)				
	Phylum	Class	Order	Family	Species	Mukgok	Jangchi	Changseon	Sagok	Songpo
Microscope	Heterokontophyta	Bacillariophyceae	Bacillariales	Bacillariaceae	*Nitzschia frustulum*	0.44	14.44	21.78	26.22	48.89
	Heterokontophyta	Bacillariophyceae	Bacillariales	Bacillariaceae	*Nitzschia inconspicua*	16.67	0.00	15.11	27.56	9.33
	Heterokontophyta	Bacillariophyceae	Bacillariales	Bacillariaceae	*Nitzschia intermedia*	0.00	33.33	11.78	0.00	0.00
	Heterokontophyta	Bacillariophyceae	Cymbellales	Gomphonemataceae	*Gomphonema* sp.	10.00	0.00	0.00	0.00	0.44
	Heterokontophyta	Bacillariophyceae	Cymbellales	Rhoicospheniaceae	*Rhoicosphenia abbreviata*	8.00	4.00	1.78	10.67	2.89
	Heterokontophyta	Bacillariophyceae	Achnanthales	Achnanthaceae	*Planothidium lanceolatum*	0.22	0.22	6.22	0.00	0.66
	Heterokontophyta	Bacillariophyceae	Naviculales	Naviculaceae	*Navicula gregaria*	5.33	0.67	10.44	10.44	26.67
	Heterokontophyta	Bacillariophyceae	Naviculales	Naviculaceae	*Navicula perminuta*	7.56	11.33	5.56	5.33	4.67
	Heterokontophyta	Bacillariophyceae	Naviculales	Naviculaceae	*Navicula recens*	2.22	18.44	0.67	10.44	0.67
	Heterokontophyta	Bacillariophyceae	Naviculales	Naviculaceae	*Navicula* sp.	11.78	0.22	0.22	0.44	0.00
	Heterokontophyta	Coscinodiscophyceae	Melosirales	Melosiraceae	*Melosira discigera*	11.33	6.00	1.55	0.00	0.00
	Heterokontophyta	Fragilariophyceae	Fragilariales	Staurosiraceae	*Staurosirella pinnata*	7.56	0.00	0.00	0.67	0.00
Total abundance of marked species in Heterokontophyta community analyzed by microscope						73.58	88.35	64.06	91.16	93.85
Illumina MiSeq	Heterokontophyta				Diatom endosymbiont	0.01	0.44	0.40	0.61	6.71
	Heterokontophyta	Bacillariophyceae	Surirellales	Entomoneidaceae	*Entomoneis paludosa*	6.85	0.00	0.00	0.00	0.00
	Heterokontophyta	Bacillariophyceae	Bacillariales	Bacillariaceae	*Bacillaria* sp.	0.00	0.00	0.66	3.80	13.14
	Heterokontophyta	Bacillariophyceae	Cymbellales	Rhoicospheniaceae	*Rhoicosphenia* sp.	0.00	0.19	0.00	0.03	7.44
	Heterokontophyta	Bacillariophyceae	Achnanthales	Achnanthaceae	*Achnanthes* sp.	0.06	6.26	1.57	0.13	0.00
	Heterokontophyta	Bacillariophyceae	Surirellales	Entomoneidaceae	*Entomoneis alata*	0.14	0.00	0.00	13.29	0.00
	Heterokontophyta	Bacillariophyceae	Naviculales	Naviculaceae	*Navicula gregaria*	0.85	7.29	3.09	18.24	25.80
	Heterokontophyta	Bacillariophyceae	Naviculales	Naviculaceae	*Navicula* sp.	1.14	5.01	1.47	23.88	8.23
	Heterokontophyta	Bacillariophyceae	Surirellales	Surirellaceae	*Surirella* sp.	0.02	0.00	0.31	6.61	0.10
	Heterokontophyta	Coscinodiscophyceae	Aulacoseirales	Aulacoseiraceae	*Aulacoseira granulata*	5.14	0.15	0.00	0.50	3.46
	Heterokontophyta	Coscinodiscophyceae	Melosirales	Melosiraceae	*Melosira arctica*	0.00	0.00	7.39	0.00	0.00
	Heterokontophyta	Coscinodiscophyceae	Melosirales	Melosiraceae	*Melosira* sp.	74.63	70.71	72.21	1.38	14.74
	Heterokontophyta	Coscinodiscophyceae	Melosirales	Melosiraceae	*Melosira varians*	0.00	1.53	0.00	0.25	8.11
Total abundance of marked species in Heterokontophyta community analyzed by Illumina MiSeq						88.84	91.58	87.10	68.72	87.73

The diatom species detected in at least one of the five samples are shown. Unclassified taxonomic names (phylum, class, order, family, and species) are replaced using underlining (__)

### Evaluation of Biological Parameters Using Microscopy and Illumina MiSeq

The biological parameters for the sampled diatom communities were calculated based on the microscopy and Illumina MiSeq results (Table 2). The two methods did not yield consistent values across all samples. In the Illumina MiSeq results, relatively high dominance values, low diversity, and evenness values were obtained from the Mukgok, Jangchi, and Changseon samples, while the results from Sagok and Songpo showed the opposite trend. These results did not show a tendency to align with richness value or number of species. The two methods obtained higher richness values from the method that detected more species. Regarding TDI and DAIpo results, similar values were obtained for microscopy and Illumina MiSeq results, especially for DAIpo; the difference between the two results for the same sample was within 3.59%.

**Table 2:** Biological parameters of Heterokontophyta community in the five estuaries

	Mukgok		Jangchi		Changseon		Sagok		Songpo	
	Microscopy	Illumina Miseq	Microscopy	Illumina Miseq	Microscopy	Illumina Miseq	Microscopy	Illumina Miseq	Microscopy	Illumina Miseq
Dominance	0.284	0.815	0.505	0.783	0.281	0.801	0.505	0.424	0.742	0.436
Diversity	2.843	1.683	3.031	1.843	4.338	1.915	2.997	3.735	2.228	3.282
Richness	7.857	4.539	2.070	4.065	4.154	3.950	2.083	4.944	1.511	3.398
Evenness	0.730	0.297	0.618	0.342	0.765	0.360	0.623	0.676	0.525	0.676
Number of species	49	51	30	42	51	40	30	46	20	29
TDI^a^	43.0	15.3	24.6	23.8	22.0	22.7	14.3	24.8	17.7	15.8
DAIpo^b^	49.9	50.0	51.9	50.1	50.9	50.0	50.8	50.0	53.3	53.2

TDI^a^: Trophic diatom index

DAIpo^b^: Diatom Assemblage Index to organic water pollution

## Discussion

The microscopy and Illumina MiSeq results showed that species belonging to specific taxa exist abundantly from the class to the family level. However, regarding the total number of taxa detected, the Illumina MiSeq analysis results were superior to those of the microscopic analysis. Exceptionally, it was possible to detect relatively more diverse species via microscopy by limiting it to the species level, although these results tended to be biased toward specific taxa at the upper taxonomic level. Based on the above findings, diatoms in microalgal communities can be effectively detected via Illumina MiSeq [13]. However, this method seems to have obvious limitations in detecting taxa at the species level [37], possibly due to the lack of registered information regarding the marker gene in each diatom species [12, 13, 37, 38]. Therefore, more accurate analyses of diatom communities can be guaranteed only when abundant information is available [12, 38, 39]. Although microscopy analysis yielded relatively abundant results for specific taxa, more taxa were identified at the species level [38–40]. Thus, this method is considered relatively effective for detecting and identifying taxa at the species level [40]. This suggests that it is relatively ineffective for analyzing communities but advantageous for collecting detailed information about diatom species [40–42]. There are commonalities between the results obtained via microscopy and Illumina MiSeq (Figs. 2, 3 and 4; Table S1). In both analyses, the abundances of species belonging to different phyla at different taxonomic levels, i.e., Bacillariophyceae (at the class level; 93.55% via microscopy and 70.79% Illumina MiSeq), Bacillariales (at the order level; microscopic: 31.18%; Illumina MiSeq: 13.48%), Naviculales (at the order level, microscopic: 27.96%; Illumina MiSeq: 34.83%), Bacillariaceae (at the family level, microscopic: 31.18%; Illumina MiSeq: 13.48%), and Naviculaceae (at the family level, microscopic: 21.51%; Illumina MiSeq: 13.48%) were high. Although both methods tended to detect similar species compositions, clear differences were noted. Illumina MiSeq analysis (which identified five classes, 21 orders, 32 families, and 88 species, including unclassified results) could detect higher diversities within each taxonomic category, from the class to the species level, compared with the microscopic analysis (which revealed three classes, 12 orders, 17 families, and 92 species, including unclassified results). Diatom species belonging to the class Mediophyceae and the orders Rhopalodiales, Chaetocerotales, Rhizosoleniales, Rhaponeidales, Stephanopyxales, Cymatosirales, Eupodiscales, and Hemiaulales could not be detected via microscopy. Although microscopy was the only method to detect the order of Licmophorales, generally speaking, from the class to the order levels, the results obtained by Illumina MiSeq were superior regarding the number of detected taxa. At the family level, the tendency of Illumina MiSeq to detect relatively higher taxonomic diversity was more pronounced. Seventeen families were detected only via Illumina MiSeq, whereas four families (Cocconeidaceae, Cymbellaceae, Thalassiosiraceae, and Ulnariaceae) were detected by microscopy. Although 13 families were commonly detected at the family level by both methods (microscopy and Illumina MiSeq analyses), only six species (*Rhoicosphenia abbreviata*, *Navicula cryptocephala*, *Navicula gregaria*, *Navicula perminuta*, *Melosira discigera*, and *Melosira varians*) were commonly detected at the species level. The above findings suggest that the lower the taxonomic level, the greater the difference between the results obtained by the two methods. Ironically, more taxa were detected by Illumina MiSeq for each taxonomic category, from the class to the family levels, but a higher number of species were identified by microscopy. The limitations of using the 18s rRNA marker in diatom identification are considered to be the cause of the poor Illumina MiSeq analysis results at the species level [43]. Previous studies found it difficult to expect perfect agreement between 18s rRNA-based classification in diatom identification [22, 43–47]. This is particularly prominent, especially for the genus *Nitzschia* [43]. Our results also showed that species belonging to the genus *Nitzschia* did not form a clade within the phylogenetic tree (Fig. 4). Therefore, the discrepancy between the results obtained from the two methods is expected to be related to the low agreement between the morphological and molecular classifications due to limitations in diatom species identification using the 18s rRNA data. Based on these results, we support Illumina MiSeq analysis as a more effective method for analyzing diatom communities. However, extensive genetic information concerning each diatom species must be available for accurate analyses via Illumina MiSeq.

The microscopic and Illumina MiSeq analyses yielded conflicting results regarding diatom classification at low taxonomic levels, such as the genus and species. Microscopy (Fig. 5; Table 1) confirmed that the genera *Nitzschia* and *Navicula* diatoms dominated at the genus level, and some species belonging to them were predominant. In contrast, Illumina MiSeq confirmed the low abundance of *Nitzschia* and the high abundance of *Melosira* and *Entomoneis* at the genus level. In addition, in many of the Illumina MiSeq results, the species name was classified as “sp.”. Based on the above, it was confirmed that microscopy can accurately identify taxonomic categories down to the species level, whereas Illumina MiSeq is limited in this area. This is possibly due to the strengths and weaknesses of the two methods [8, 12, 23, 38, 40]. Although identification through microscopic observation can vary greatly depending on the skill and experience of the observer, it can provide accurate identification at the species level [41, 42]. Conversely, Illumina MiSeq, which depends on the target sequence for identification, may interpret data differently depending on the database being used as the reference [13, 48]. However, although detailed identification of each diatom cell is important, sample size must also be considered to obtain reliable results when analyzing diatom communities [10, 38]. One of the reasons why microscopy is limited in the analysis of communities (even though species-level identification is possible) is that substantial human resources and time are required to process samples [8, 12]. Moreover, the results can be fatally biased because microscopy is applied to relatively small samples [12, 41]. Illumina MiSeq is unaffected by these issues and can efficiently analyze relatively large samples in a relatively short time; consequently, this method is superior to microscopy in the analysis of microbial communities [12, 13]. Although microscopy allows accurate identification, it has obvious limitations in analyzing communities. Therefore, it is necessary to use Illumina MiSeq to analyze many samples efficiently. However, to improve the accuracy of the Illumina MiSeq results, a precise and extensive database containing molecular identification keys is required; meanwhile, microscopy can significantly contribute to creating such databases.

In previous research, microscopy and Illumina MiSeq analyses must be improved to evaluate the diatom community [49, 50]. For microscopy-dependent analysis, the minimum number of valve settings affects the results. In our study, 450 valves were set as a minimum, although this number may have limited the detection of rare species [49, 51]. Limited detection of rare species in the community may have contributed to the gap between the microscopy and Illumina MiSeq analyses [49, 51]. In this regard, Illumina MiSeq can reduce the possibility of missing taxa by identifying the relationship between the number of reads and OTUs using rarefaction curves [52]. Moreover, using updated identification keys and the reflection of reclassified taxa can be major variables in using microscopy [53, 54]. Conversely, the reference database can significantly affect Illumina MiSeq analyses [50]. In a previous study, the V4 and V9 regions in the 18s rRNA were compared, and a difference was identified between the two results obtained from the same sample [50]. The completeness of the reference database is an important factor in Illumina MiSeq analyses, and there is a possibility that the same data may show different results [50]. In our results, there is a clear difference in the results obtained from the two methodologies, and the above variables should be considered regarding the differences between the results.

The biological parameters calculated based on the microscopy and Illumina MiSeq analyses confirmed that the two methods yielded inconsistent results for all parameters; in any case, they did not converge to 100%, even though the same samples were analyzed. High dominance values were accompanied by low diversity and evenness values, and results with a high number of species tended to be accompanied by a high richness value [55–57]. Moreover, the correlation between the microscopy and Illumina MiSeq results of the biological parameters (dominance, diversity, and evenness; number of species and richness) was not observed. On the other hand, a particular regularity could be easily found between the TDI and DAIpo values [58, 59]. Although both methods employed regular detections between the calculated biological parameters, the clear differences observed between the results obtained from the same samples indicated that the effectiveness of these methods needs to be evaluated [56, 58]. In the analysis of diatom communities, either method could contain limitations, or both methodologies (microscopy, Illumina MiSeq) may have significant limitations [12]. Accurate diatom community analysis results must be the basis to accurately analyze diatom communities using biological parameters, such as dominance, diversity, richness, evenness, TDI, and DAIpo [55–59]. Therefore, it is important to evaluate the effectiveness of each method and suggest solutions to their limitations.

Microscopy and Illumina MiSeq were used to analyze diatom communities effectively, and the results were compared (Figs. 2, 3, 4 and 5; Tables 1 and 2 and Table S1). The results obtained at the species level (the lowest taxonomic level) generally showed a clearer difference compared with those at the class level (the upper taxonomic level) [8, 10, 12]. Furthermore, the results obtained by microscopy showed high taxonomy discrimination, which provided the basis to use microscopy to research the diatom communities [9, 40, 42]. In addition, microscopy requires no special materials other than a microscope and sample preparation at a relatively low cost [42]. However, the number of experts is limited and currently declining, which directly decreases the reliability of the results obtained through microscopy [12, 60]. Furthermore, extensive human resources and time are required to obtain adequate data [12, 61]. In contrast, Illumina MiSeq is less affected by these limitations, which is one of the reasons why it is used to analyze diatom communities [39, 60]. Illumina MiSeq analysis does not require skilled observers; this method is available to researchers who are inexperienced in taxonomic identification [11, 13, 39]. Furthermore, Illumina MiSeq can effectively process large amounts of data in a relatively short time [13, 37]. However, there is a limitation, whereby the results can be affected by the process of preparing samples for analysis and the quality of the reference database [13, 23, 40, 48]. Therefore, Illumina MiSeq is undoubtedly one of the most powerful tools, although it can be risky to analyze communities exclusively using Illumina MiSeq [12, 39, 40]. Microscopy and Illumina MiSeq can complement each other because each method has strengths that can compensate for the limitations of the other [38, 48]. This can be accomplished by constructing a high-quality database based on accurate taxonomy information obtained through microscopy and analyzing diatom communities more effectively via Illumina MiSeq using the improved database.

## Conclusion

In this study, microscopy and Illumina MiSeq were compared in terms of their effectiveness in analyzing diatom communities. Specifically, the results obtained from each method were compared regarding the taxonomic identification of diatoms in the communities. Furthermore, it was confirmed that the two methods can effectively analyze the structural characteristics of communities. Although microscopy was excellent in revealing the taxonomic identity of diatoms, it had clear limitations in evaluating the characteristics of communities due to the small number of samples analyzed. Conversely, Illumina MiSeq can effectively process a large amount of data and be more successfully applied to diatom community analyses. However, to ensure its effectiveness, the support of a high-quality database is essential. In conclusion, microscopy and Illumina MiSeq have distinct strengths and weaknesses, and neither is a perfect solo method. Therefore, an appropriate method should be selected according to the characteristics of the study, and it is recommended that microscopy be applied for phylogenetic and floristic purposes and that the Illumina MiSeq be used for fast biomonitoring or detection of diatoms. Furthermore, both methods must be improved for effective research on diatom communities to effectively identify and classify multiple samples for microscopic methods and construct an accurate and extensive database for Illumina MiSeq analysis. This study suggests that improvements in classical and molecular methods for studying diatom communities can be achieved through a combination of microscopy and Illumina MiSeq analyses.

## Supplementary Information

Below is the link to the electronic supplementary material.

ESM 1 (DOCX 45.1 KB)

## Data Availability

No datasets were generated or analysed during the current study.
