# Supplementary material for: Comparison of Metabarcoding and Microscopy Methodologies to Analyze Diatom Communities in Five Estuaries Along the Southern Coast of the Korean Peninsula
Source: Microb Ecol. 2024 Jul 17;87(1):95. doi: 10.1007/s00248-024-02396-x (PMC11255046; doi:10.1007/s00248-024-02396-x)
Supplement: Supplementary file 1 — (DOCX 45.1 KB) [file 248_2024_2396_MOESM1_ESM.docx]

**Table S1.** Taxonomy of Heterokontophyta species detected by microscopy and Illumina MiSeq.

| Method | Taxonomy | | | | |
| --- | --- | --- | --- | --- | --- |
|  | Phylum | Class | Order | Family | Species |
| Microscope | Heterokontophyta | Bacillariophyceae | Surirellales | Entomoneidaceae | *Entomoneis alata* |
|  | Heterokontophyta | Bacillariophyceae | Bacillariales | Bacillariaceae | *Bacillaria paxillifera* |
|  | Heterokontophyta | Bacillariophyceae | Bacillariales | Bacillariaceae | *Nitzschia acicularis* |
|  | Heterokontophyta | Bacillariophyceae | Bacillariales | Bacillariaceae | *Nitzschia aequorea* |
|  | Heterokontophyta | Bacillariophyceae | Bacillariales | Bacillariaceae | *Nitzschia amphibia* |
|  | Heterokontophyta | Bacillariophyceae | Bacillariales | Bacillariaceae | *Tryblionella coarctata* |
|  | Heterokontophyta | Bacillariophyceae | Bacillariales | Bacillariaceae | *Nitzschia communis* |
|  | Heterokontophyta | Bacillariophyceae | Bacillariales | Bacillariaceae | *Tryblionella apiculata* |
|  | Heterokontophyta | Bacillariophyceae | Bacillariales | Bacillariaceae | *Nitzschia debilis* |
|  | Heterokontophyta | Bacillariophyceae | Bacillariales | Bacillariaceae | *Nitzschia dissipata* |
|  | Heterokontophyta | Bacillariophyceae | Bacillariales | Bacillariaceae | *Nitzschia filiformis* |
|  | Heterokontophyta | Bacillariophyceae | Bacillariales | Bacillariaceae | *Nitzschia fonticola* |
|  | Heterokontophyta | Bacillariophyceae | Bacillariales | Bacillariaceae | *Nitzschia frustulum* |
|  | Heterokontophyta | Bacillariophyceae | Bacillariales | Bacillariaceae | *Tryblionella hungarica* |
|  | Heterokontophyta | Bacillariophyceae | Bacillariales | Bacillariaceae | *Nitzschia hybrida* |
|  | Heterokontophyta | Bacillariophyceae | Bacillariales | Bacillariaceae | *Nitzschia inconspicua* |
|  | Heterokontophyta | Bacillariophyceae | Bacillariales | Bacillariaceae | *Nitzschia intermedia* |
|  | Heterokontophyta | Bacillariophyceae | Bacillariales | Bacillariaceae | *Nitzschia lanceola* |
|  | Heterokontophyta | Bacillariophyceae | Bacillariales | Bacillariaceae | *Tryblionella levidensis* |
|  | Heterokontophyta | Bacillariophyceae | Bacillariales | Bacillariaceae | *Nitzschia linearis* |
|  | Heterokontophyta | Bacillariophyceae | Bacillariales | Bacillariaceae | *Tryblionella littoralis* |
|  | Heterokontophyta | Bacillariophyceae | Bacillariales | Bacillariaceae | *Nitzschia palea* |
|  | Heterokontophyta | Bacillariophyceae | Bacillariales | Bacillariaceae | *Nitzschia perminuta* |
|  | Heterokontophyta | Bacillariophyceae | Bacillariales | Bacillariaceae | *Nitzschia pusilla* |
|  | Heterokontophyta | Bacillariophyceae | Bacillariales | Bacillariaceae | *Nitzschia recta* |
|  | Heterokontophyta | Bacillariophyceae | Bacillariales | Bacillariaceae | *Nitzschia sigma* |
|  | Heterokontophyta | Bacillariophyceae | Bacillariales | Bacillariaceae | *Nitzschia sigmoidea* |
|  | Heterokontophyta | Bacillariophyceae | Bacillariales | Bacillariaceae | *Nitzschia* sp. |
|  | Heterokontophyta | Bacillariophyceae | Bacillariales | Bacillariaceae | *Nitzschia tubicola* |
|  | Heterokontophyta | Bacillariophyceae | Bacillariales | Bacillariaceae | *Nitzschia valdestriata* |
|  | Heterokontophyta | Bacillariophyceae | Achnanthales | Cocconeidaceae | *Cocconeis placentula* |
|  | Heterokontophyta | Bacillariophyceae | Achnanthales | Cocconeidaceae | *Cocconeis scutellum* |
|  | Heterokontophyta | Bacillariophyceae | Achnanthales | Cocconeidaceae | *Cocconeis* sp. |
|  | Heterokontophyta | Bacillariophyceae | Cymbellales | Cymbellaceae | *Cymbella tumida* |
|  | Heterokontophyta | Bacillariophyceae | Cymbellales | Cymbellaceae | *Cymbella turgidula* |
|  | Heterokontophyta | Bacillariophyceae | Cymbellales | Gomphonemataceae | *Gomphonema gracile* |
|  | Heterokontophyta | Bacillariophyceae | Cymbellales | Gomphonemataceae | *Gomphonema parvulum* |
|  | Heterokontophyta | Bacillariophyceae | Cymbellales | Gomphonemataceae | *Gomphonema* sp. |
|  | Heterokontophyta | Bacillariophyceae | Cymbellales | Rhoicospheniaceae | *Rhoicosphenia abbreviata* |
|  | Heterokontophyta | Bacillariophyceae | Achnanthales | Achnanthaceae | *Karayevia amoena* |
|  | Heterokontophyta | Bacillariophyceae | Achnanthales | Achnanthaceae | *Achnanthes adnata* |
|  | Heterokontophyta | Bacillariophyceae | Achnanthales | Achnanthidiaceae | *Planothidium delicatulum* |
|  | Heterokontophyta | Bacillariophyceae | Achnanthales | Achnanthidiaceae | *Planothidium lanceolatum* |
|  | Heterokontophyta | Bacillariophyceae | Achnanthales | Achnanthidiaceae | *Achnanthidium minutissimum* |
|  | Heterokontophyta | Bacillariophyceae | Naviculales | Berkeleyaceae | *Berkeleya rutilans* |
|  | Heterokontophyta | Bacillariophyceae | Naviculales | Diploneidaceae | *Diploneis oblongella* |
|  | Heterokontophyta | Bacillariophyceae | Naviculales | Diploneidaceae | *Diploneis parma* |
|  | Heterokontophyta | Bacillariophyceae | Naviculales | Diploneidaceae | *Diploneis subovalis* |
|  | Heterokontophyta | Bacillariophyceae | Naviculales | Naviculaceae | *Navicula arenaria* |
|  | Heterokontophyta | Bacillariophyceae | Naviculales | Naviculaceae | *Mayamaea atomus* |
|  | Heterokontophyta | Bacillariophyceae | Naviculales | Sellaphoraceae | *Sellaphora bacillum* |
|  | Heterokontophyta | Bacillariophyceae | Naviculales | Naviculaceae | *Navicula capitata* |
|  | Heterokontophyta | Bacillariophyceae | Naviculales | Diadesmidaceae | *Humidophila contenta* |
|  | Heterokontophyta | Bacillariophyceae | Naviculales | Naviculaceae | *Navicula cryptocephala* |
|  | Heterokontophyta | Bacillariophyceae | Naviculales | Naviculaceae | *Navicula gregaria* |
|  | Heterokontophyta | Bacillariophyceae | Naviculales | Naviculaceae | *Navicula hintzii* |
|  | Heterokontophyta | Bacillariophyceae | Naviculales | Naviculaceae | *Navicula peregrina* |
|  | Heterokontophyta | Bacillariophyceae | Naviculales | Naviculaceae | *Navicula perminuta* |
|  | Heterokontophyta | Bacillariophyceae | Naviculales | Naviculaceae | *Navicula recens* |
|  | Heterokontophyta | Bacillariophyceae | Naviculales | Naviculaceae | *Navicula rhynchocephala* |
|  | Heterokontophyta | Bacillariophyceae | Naviculales | Naviculaceae | *Navicula salinarum* |
|  | Heterokontophyta | Bacillariophyceae | Naviculales | Naviculaceae | *Navicula* sp. |
|  | Heterokontophyta | Bacillariophyceae | Naviculales | Stauroneidaceae | *Craticula subminuscula* |
|  | Heterokontophyta | Bacillariophyceae | Naviculales | Naviculaceae | *Navicula tenelloides* |
|  | Heterokontophyta | Bacillariophyceae | Naviculales | Naviculaceae | *Pseudofallacia tenera* |
|  | Heterokontophyta | Bacillariophyceae | Naviculales | Naviculaceae | *Navicula tripunctata* |
|  | Heterokontophyta | Bacillariophyceae | Naviculales | Naviculaceae | *Navicula zanonii* |
|  | Heterokontophyta | Bacillariophyceae | Naviculales | Naviculaceae | *Gyrosigma fasciola* |
|  | Heterokontophyta | Bacillariophyceae | Naviculales | Pleurosigmataceae | *Pleurosigma elongatum* |
|  | Heterokontophyta | Bacillariophyceae | Surirellales | Surirellaceae | *Surirella brebissonii* |
|  | Heterokontophyta | Bacillariophyceae | Surirellales | Surirellaceae | *Surirella minuta* |
|  | Heterokontophyta | Bacillariophyceae | Naviculales | Amphipleuraceae | *Halamphora coffeiformis* |
|  | Heterokontophyta | Bacillariophyceae | Thalassiophysales | Catenulaceae | *Amphora copulata* |
|  | Heterokontophyta | Bacillariophyceae | Naviculales | Amphipleuraceae | *Halamphora holsatica* |
|  | Heterokontophyta | Bacillariophyceae | Thalassiophysales | Catenulaceae | *Amphora pediculus* |
|  | Heterokontophyta | Bacillariophyceae | Thalassiophysales | Catenulaceae | *Amphora* sp. |
|  | Heterokontophyta | Bacillariophyceae | Naviculales | Naviculaceae | *Seminavis strigosa* |
|  | Heterokontophyta | Coscinodiscophyceae | Melosirales | Melosiraceae | *Melosira discigera* |
|  | Heterokontophyta | Coscinodiscophyceae | Melosirales | Melosiraceae | *Melosira varians* |
|  | Heterokontophyta | Mediophyceae | Thalassiosirales | Thalassiosiraceae | *Stephanocyclus meneghinianus* |
|  | Heterokontophyta | Mediophyceae | Stephanodiscales | Stephanodiscaceae | *Discostella stelligera* |
|  | Heterokontophyta | Mediophyceae | Thalassiosirales | Thalassiosiraceae | *Thalassiosira bramaputrae* |
|  | Heterokontophyta | Mediophyceae | Thalassiosirales | Thalassiosiraceae | *Conticribra weissflogii* |
|  | Heterokontophyta | Bacillariophyceae | Fragilariales | Fragilariaceae | *Fragilaria rhabdosoma* |
|  | Heterokontophyta | Bacillariophyceae | Fragilariales | Fragilariaceae | *Fragilaria capucina* |
|  | Heterokontophyta | Bacillariophyceae | Fragilariales | Staurosiraceae | *Staurosira construens* |
|  | Heterokontophyta | Bacillariophyceae | Fragilariales | Fragilariaceae | *Jousea elliptica* |
|  | Heterokontophyta | Bacillariophyceae | Licmophorales | Ulnariaceae | *Tabularia fasciculata* |
|  | Heterokontophyta | Bacillariophyceae | Fragilariales | Staurosiraceae | *Staurosirella pinnata* |
|  | Heterokontophyta | Bacillariophyceae | Rhabdonematales | Tabellariaceae | *Meridion* sp. |
|  | Heterokontophyta | Bacillariophyceae | Licmophorales | Ulnariaceae | *Ctenophora pulchella* |
|  | Heterokontophyta | Bacillariophyceae | Licmophorales | Ulnariaceae | *Ulnaria ulna* |
| Illumina MiSeq | Heterokontophyta | __ | __ | __ | *Diatom endosymbiont* |
|  |  |  |  |  |  |
|  | Heterokontophyta | Bacillariophyceae | Surirellales | Entomoneidaceae | *Entomoneis paludosa* |
|  | Heterokontophyta | Bacillariophyceae | Surirellales | Entomoneidaceae | *Entomoneis* sp. |
|  | Heterokontophyta | Bacillariophyceae | Bacillariales | Bacillariaceae | *Bacillaria* sp. |
|  | Heterokontophyta | Bacillariophyceae | Bacillariales | Bacillariaceae | *Denticula kuetzingii* |
|  | Heterokontophyta | Bacillariophyceae | Bacillariales | Bacillariaceae | *Nitzschia biundulata* |
|  | Heterokontophyta | Bacillariophyceae | Bacillariales | Bacillariaceae | *Nitzschia capitellata* |
|  | Heterokontophyta | Bacillariophyceae | Bacillariales | Bacillariaceae | *Nitzschia longissima* |
|  | Heterokontophyta | Bacillariophyceae | Bacillariales | Bacillariaceae | *Nitzschia navis-varingica* |
|  | Heterokontophyta | Bacillariophyceae | Bacillariales | Bacillariaceae | *Nitzschia reskoi* |
|  | Heterokontophyta | Bacillariophyceae | Bacillariales | Bacillariaceae | *Nitzschia* sp. |
|  | Heterokontophyta | Bacillariophyceae | Bacillariales | Bacillariaceae | *Nitzschia supralitorea* |
|  | Heterokontophyta | Bacillariophyceae | Bacillariales | Bacillariaceae | *Psammodictyon* sp. |
|  | Heterokontophyta | Bacillariophyceae | Bacillariales | Bacillariaceae | *Pseudo-nitzschia americana* |
|  | Heterokontophyta | Bacillariophyceae | Bacillariales | Bacillariaceae | *Tryblionella gaoana* |
|  | Heterokontophyta | Bacillariophyceae | Achnanthales | Achnanthidiaceae | *Achnanthidium saprophilum* |
|  | Heterokontophyta | Bacillariophyceae | Achnanthales | Achnanthidiaceae | *Astartiella* sp. |
|  | Heterokontophyta | Bacillariophyceae | Cymbellales | Gomphonemataceae | *Gomphonema acuminatum* |
|  | Heterokontophyta | Bacillariophyceae | Cymbellales | Rhoicospheniaceae | *Rhoicosphenia abbreviata* |
|  | Heterokontophyta | Bacillariophyceae | Cymbellales | Rhoicospheniaceae | *Rhoicosphenia* sp. |
|  | Heterokontophyta | Bacillariophyceae | Achnanthales | Achnanthaceae | *Achnanthes* sp. |
|  | Heterokontophyta | Bacillariophyceae | Achnanthales | Achnanthaceae | *Planothidium* sp. |
|  | Heterokontophyta | Bacillariophyceae | Achnanthales | Achnanthaceae | *Planothidium suncheonmanense* |
|  | Heterokontophyta | Bacillariophyceae | Naviculales | Naviculaceae | *Navicula* sp. |
|  | Heterokontophyta | Bacillariophyceae | Surirellales | Entomoneidaceae | *Entomoneis alata* |
|  | Heterokontophyta | Bacillariophyceae | Naviculales | Amphipleuraceae | *Halamphora adumbratoides* |
|  | Heterokontophyta | Bacillariophyceae | Naviculales | Amphipleuraceae | *Halamphora bicapitata* |
|  | Heterokontophyta | Bacillariophyceae | Naviculales | Amphipleuraceae | *Halamphora halophila* |
|  | Heterokontophyta | Bacillariophyceae | Naviculales | Amphipleuraceae | *Halamphora pertusa* |
|  | Heterokontophyta | Bacillariophyceae | Naviculales | Amphipleuraceae | *Halamphora pseudoholsatica* |
|  | Heterokontophyta | Bacillariophyceae | Naviculales | Amphipleuraceae | *Halamphora* sp. |
|  | Heterokontophyta | Bacillariophyceae | Naviculales | Amphipleuraceae | *Halamphora subtropica* |
|  | Heterokontophyta | Bacillariophyceae | Naviculales | Amphipleuraceae | *Halamphora tenucostata* |
|  | Heterokontophyta | Bacillariophyceae | Naviculales | Diadesmidaceae | *Humidophila gallica* |
|  | Heterokontophyta | Bacillariophyceae | Naviculales | Diadesmidaceae | *Luticola permuticopsis* |
|  | Heterokontophyta | Bacillariophyceae | Naviculales | Diploneidaceae | *Diploneis* sp. |
|  | Heterokontophyta | Bacillariophyceae | Naviculales | Diploneidaceae | *Diploneis* sp. |
|  | Heterokontophyta | Bacillariophyceae | Naviculales | Naviculaceae | *Caloneis silicula* |
|  | Heterokontophyta | Bacillariophyceae | Naviculales | Stauroneidaceae | *Fistulifera saprophila* |
|  | Heterokontophyta | Bacillariophyceae | Naviculales | Naviculaceae | *Haslea nipkowii* |
|  | Heterokontophyta | Bacillariophyceae | Naviculales | Naviculaceae | *Hippodonta capitata* |
|  | Heterokontophyta | Bacillariophyceae | Naviculales | Naviculaceae | *Mayamaea permitis* |
|  | Heterokontophyta | Bacillariophyceae | Naviculales | Naviculaceae | *Navicula cryptocephala* |
|  | Heterokontophyta | Bacillariophyceae | Naviculales | Naviculaceae | *Navicula gregaria* |
|  | Heterokontophyta | Bacillariophyceae | Naviculales | Naviculaceae | *Navicula perminuta* |
|  | Heterokontophyta | Bacillariophyceae | Naviculales | Naviculaceae | *Navicula salinicola* |
|  | Heterokontophyta | Bacillariophyceae | Naviculales | Naviculaceae | *Navicula* sp. |
|  | Heterokontophyta | Bacillariophyceae | Naviculales | Naviculaceae | *Navicula trivialis* |
|  | Heterokontophyta | Bacillariophyceae | Naviculales | Naviculaceae | *Navicula veneta* |
|  | Heterokontophyta | Bacillariophyceae | Naviculales | Pleurosigmataceae | *Pleurosigma intermedium* |
|  | Heterokontophyta | Bacillariophyceae | Naviculales | Pleurosigmataceae | *Pleurosigma* sp. |
|  | Heterokontophyta | Bacillariophyceae | Naviculales | Sellaphoraceae | *Sellaphora laevissima* |
|  | Heterokontophyta | Bacillariophyceae | Naviculales | Stauroneidaceae | *Stauroneis kriegeri* |
|  | Heterokontophyta | Bacillariophyceae | Naviculales | Stauroneidaceae | *Stauroneis latistauros* |
|  | Heterokontophyta | Bacillariophyceae | Rhopalodiales | Rhopalodiaceae | *Epithemia argus* |
|  | Heterokontophyta | Bacillariophyceae | Surirellales | Surirellaceae | *Surirella* sp. |
|  | Heterokontophyta | Bacillariophyceae | Thalassiophysales | Catenulaceae | *Amphora* sp. |
|  | Heterokontophyta | Coscinodiscophyceae | Aulacoseirales | Aulacoseiraceae | *Aulacoseira granulata* |
|  | Heterokontophyta | Mediophyceae | Chaetocerotales | Chaetocerotaceae | *Chaetoceros calcitrans* |
|  | Heterokontophyta | Mediophyceae | Chaetocerotales | Chaetocerotaceae | *Chaetoceros constrictus* |
|  | Heterokontophyta | Mediophyceae | Chaetocerotales | Chaetocerotaceae | *Chaetoceros curvisetus* |
|  | Heterokontophyta | Mediophyceae | Chaetocerotales | Chaetocerotaceae | *Chaetoceros rotosporus* |
|  | Heterokontophyta | Mediophyceae | Chaetocerotales | Chaetocerotaceae | *Chaetoceros* sp. |
|  | Heterokontophyta | Mediophyceae | Chaetocerotales | Chaetocerotaceae | *Chaetoceros tenuissimus* |
|  | Heterokontophyta | Coscinodiscophyceae | Melosirales | Melosiraceae | *Melosira arctica* |
|  | Heterokontophyta | Coscinodiscophyceae | Melosirales | Melosiraceae | *Melosira discigera* |
|  | Heterokontophyta | Coscinodiscophyceae | Melosirales | Melosiraceae | *Melosira* sp. |
|  | Heterokontophyta | Coscinodiscophyceae | Melosirales | Melosiraceae | *Melosira varians* |
|  | Heterokontophyta | Coscinodiscophyceae | Stephanopyxales | Stephanopyxidaceae | *Eupyxidicula turris* |
|  | Heterokontophyta | Coscinodiscophyceae | Rhizosoleniales | Rhizosoleniaceae | *Guinardia delicatula* |
|  | Heterokontophyta | Mediophyceae | Thalassiosirales | Skeletonemataceae | *Skeletonema costatum* |
|  | Heterokontophyta | Mediophyceae | Thalassiosirales | Skeletonemataceae | *Skeletonema menzelii* |
|  | Heterokontophyta | Mediophyceae | Thalassiosirales | Skeletonemataceae | *Skeletonema* sp. |
|  | Heterokontophyta | Mediophyceae | Stephanodiscales | Stephanodiscaceae | *Discostella lacus-karluki* |
|  | Heterokontophyta | Mediophyceae | Thalassiosirales | Thalassiosiraceae | *Conticribra guillardii* |
|  | Heterokontophyta | Mediophyceae | Thalassiosirales | Thalassiosiraceae | *Conticribra weissflogiopsis* |
|  | Heterokontophyta | Mediophyceae | Thalassiosirales | Thalassiosiraceae | *Mediolabrus comicus* |
|  | Heterokontophyta | Mediophyceae | Thalassiosirales | Thalassiosiraceae | *Thalassiosira aestivalis* |
|  | Heterokontophyta | Mediophyceae | Thalassiosirales | Thalassiosiraceae | *Thalassiosira* sp. |
|  | Heterokontophyta | Bacillariophyceae | Rhaphoneidales | Asterionellopsidaceae | *Asterionellopsis glacialis* |
|  | Heterokontophyta | Bacillariophyceae | Fragilariales | Fragilariaceae | *Gedaniella flavovirens* |
|  | Heterokontophyta | Bacillariophyceae | Fragilariales | Staurosiraceae | *Stauroforma rinceana* |
|  | Heterokontophyta | Bacillariophyceae | Rhaponeidales | Rhaphoneidaceae | *Neodelphineis* sp. |
|  | Heterokontophyta | Coscinodiscophyceae | Stephanopyxales | Hydroseraceae | *Hydrosera sp.* |
|  | Heterokontophyta | Mediophyceae | Cymatosirales | Cymatosiraceae | *Minutocellus polymorphus* |
|  | Heterokontophyta | Mediophyceae | Eupodiscales | Odontellaceae | *Odontella* sp. |
|  | Heterokontophyta | Mediophyceae | Hemiaulales | Hemiaulaceae | *Cerataulina pelagica* |

Unclassified taxonomic names (phylum, class, order, family, and species) are replaced using underlining (__).
